# Adaptive Immune System in Hypertension: Implicating the Role of T Cells

**DOI:** 10.2174/011573403X387039251002064044

**Published:** 2025-10-20

**Authors:** Padmaja Varadarajan, Sugin Lal Jabaris S, Venkataraman Krishnamurthy, Hemalatha Kanagarajan

**Affiliations:** 1 Department of Pharmacology, Siddha Central Research Institute, Central Council for Research in Siddha, Ministry of Ayush, Govt of India, Anna Govt. Hosp. Campus, Chennai, Tamil Nadu, India;; 2 Department of Pharmacology, Saveetha College of Pharmacy, Saveetha Institute of Medical and Technical Sciences, Thandalam, Chennai, Tamil Nadu, India;; 3 Department of Pharmaceutical Chemistry, Saveetha College of Pharmacy, Saveetha Institute of Medical and Technical Sciences, Thandalam, Chennai, Tamil Nadu, India

**Keywords:** Hypertension, immune system, T Cells, cytokines, endothelial dysfunction, renal inflammation

## Abstract

**Introduction:**

Emerging scientific evidence supports the essential role of inflammation as one of the factors in the development of hypertension. The immune system plays a crucial role in the body's defense mechanism and in promoting tissue regeneration after injury. When the immune system overreacts to an insult or injury, collateral damage can result in hypertension.

**Methods:**

A comprehensive literature search was conducted to collect and analyse articles related to the role of the adaptive immune system, particularly T cells and their cytokines. It was conducted in scientific databases, including PubMed, ScienceDirect, and Google Scholar, to identify relevant articles published between 2000 and 2024, ensuring an up-to-date collection of scientific evidence. The following keywords and Medical Subject Headings (MeSH terms) were used: hypertension, immune system, T cells, cytokines, IL-10, TNF-α, IL-17, IFN-γ, and renal inflammation. These keywords helped capture both basic mechanisms and clinical findings relevant to the immune-mediated mechanism of hypertension. Only peer-reviewed original research articles and review articles in English were included. Preference was given to articles focused on cytokine synthesis, expression, release, and their signalling pathways, as well as hypertension-related target organ damage, especially in the kidney and blood vessels. All the articles were thoroughly analysed to extract information on the involvement of T cell–derived cytokines in promoting immune activation, monocyte infiltration, sodium retention, vascular dysfunction, and endothelial impairment, which all contribute to the pathogenesis of hypertension.

**Results:**

According to the reference articles, TNF-α, IL-17, and IFN-γ stimulate vascular inflammation, sodium retention, endothelial dysfunction, and renal inflammation.

**Discussion:**

The roles of T cells and the associated cytokines in regulating blood pressure are thoroughly discussed. It can activate oxidative stress and result in renal sodium imbalance, as well as interaction with the RAAS system and oxidative stress pathway, culminating in the exacerbation of blood pressure.

**Conclusion:**

It is concluded that cytokines play a significant role in the development of hypertension, and targeting them might be a plausible pharmacological intervention for managing blood pressure.

## 1 INTRODUCTION

Hypertension (HTN) is a condition characterized by chronically elevated blood pressure, with stage I defined as 130-139/80-89 mmHg and stage II defined as ≥140/≥ 90 mmHg [1]. It is a global pandemic affecting over one billion individuals [2]. Approximately 25-43% of the world population has been afflicted with hypertension [3]. A 3% decrease in the global prevalence of hypertension was observed in 2019 compared to 2010, with a focus on the voluntary global target of 21% [4]. It is a compelling cause of cardiac disorders such as stroke, myocardial infarction, congestive heart failure, and renal insufficiency. The stimuli responsible for the development of HTN are not only the sympathetic–adrenal medullary system and vasomodulatory substances such as angiotensin (Ang) II, endothelin (ET)-1, and aldosterone, but also specific mutations or the presence of gene polymorphisms. Excessive salt consumption is a significant factor in the development of elevated blood pressure (BP) [5, 6]. The heart pumps blood to ensure the circulation of gases and nutrients. The blood vessels may narrow or widen to control blood flow, and the kidneys manage the excretion of sodium and water. These three organs are fundamental in determining an individual’s blood pressure [7, 8].

Growing evidence suggests that alterations in the immune system play an essential role in the progression of hypertension. The accumulation of immune cells in blood vessels, kidneys, heart, and brain facilitates a chronic inflammatory response that disrupts the blood pressure–regulating mechanism, leading to hypertension [8]. Over half a century ago, a causal association between the immune system and hypertension was demonstrated when autoantibodies were detected in artery specimens from ‘hypertensive cadavers’ [9]. A decade later, research in this direction revealed elevated levels of circulating immunoglobulin G (IgG) in patients with high blood pressure relative to those with normal blood pressure, a discovery validated by subsequent clinical investigations [10, 11].

In recent years, with more research results unravelling, it has become perceptible that hypertension is characterized by inflammation, which includes the migration and congregation of both natural and acquired immune cells within the tissues. These cells discharge cytokines and promote oxidative stress, contributing to the pathogenesis of the condition. In HTN, the inflammatory mechanism, which encompasses adhesion protein and chemokine secretion, immune cell recruitment and penetration, cytokine liberation, and oxidative stress, appears to be at elevated levels [6]. Hypertension is consistently characterized by the penetration of non-specific immune cells (monocytes/macrophages) and specific immune cells (T lymphocytes) into perivascular adipose tissue, kidneys, and myocardium. This condition is also characterized by the increased expression of cellular adhesive proteins and chemokines, enhanced cytokine formation and secretion, and the generation of reactive oxygen species (ROS) [12, 13].

Despite significant advancements in hypertension research, the role of T cells in the development of hypertension remains to be unraveled [14]. In this review, the existing information and mechanisms behind immune cell activation and their contribution to elevated blood pressure are discussed.

## 2 METHODOLOGY

The search was carried out using scientific databases like PubMed, ScienceDirect, and Google Scholar to collect relevant articles published between 2000 and 2024 to ensure an up-to-date collection of scientific evidence. The following keywords were used: hypertension, immune system, T cells, cytokines, IL-10, TNF-α, IL-17, IFN-γ, renal inflammation, cytokine synthesis, expression, release, and their signalling pathways, as well as hypertension-related target organ damage, especially the kidney and blood vessels. This approach helped identify relevant articles related to the immune-mediated mechanism of hypertension. All the articles were thoroughly analysed to extract information on the involvement of T cell-derived cytokines in promoting immune activation, monocyte infiltration, sodium retention, and endothelial dysfunction, which play a vital role in the development and progression of hypertension.

### 2.1 Immune System

The primary defense against foreign invading microbes is categorized into two immune responses: the innate immune defense and the adaptive immune defense [15]. The two systems differ in response time, duration, functional cell types, and specificity for various categories of invading microorganisms. The innate immune response is non-specific against potentially hazardous foreign particles, whereas the adaptive immune system exhibits a profound level of recognition and specificity [16].

### 2.2 Innate Immune System

It includes epithelial cells forming a protective barrier against invasion, phagocytic cells for engulfing and eliminating external microorganisms, and the complement system, which aids in terminating and removing pathogens. The cells of this system possess pattern recognition receptors that identify and bind pathogen-associated molecular patterns, such as lipopolysaccharide, lipoteichoic acid, flagellin, double-stranded RNA, and unmethylated cytosine–guanine dinucleotides with phosphodiester linkage. These trigger a cascade of metabolic processes, including the release of cytokines and chemokines, activation of nitric oxide synthase, and the generation of reactive oxygen species [16].

Innate immune cells have a significant role in initiating and sustaining the adaptive immune response, which is an essential process in the development of hypertension and blood vessel disruption. These cells are characterized by their rapid response and are rightly called the first line of defense. Pattern recognition receptors (PRRs), like toll-like receptors (TLRs), present in these cells are capable of recognizing pathogen-associated molecular patterns (PAMPs) or damage-associated molecular patterns (DAMPs), including neoantigens from stressed or injured tissue. Innate immune responses utilize specialized functional cells to initiate host defense and inflammation, either by directly secreting cytokines and chemokines, activating complement proteins, and performing phagocytosis, or by engaging the adaptive immune system through antigen presentation [13].

### 2.3 Adaptive Immune System

The innate immune response activates the adaptive immune system. The constituents of the adaptive immune system respond more slowly but with greater specificity, leading to a stronger secondary response. The adaptive immune system often utilizes cells and molecules from the innate system to combat harmful foreign invaders. It is classified into two types: (1) B cell–mediated (humoral/antibody-mediated) and (2) T cell–mediated (cell-mediated) immune responses [16].

### 2.4 Cell-mediated Immune System

The cell-mediated immune system is an essential part of the body’s immune system; antibodies are not involved, and instead, it induces phagocytes and antigen-targeted killer T lymphocytes. These cells release various cytokines in response to an antigen attack. This system is responsible for recognizing and eliminating infected cells, cancer cells, and foreign pathogens. It is especially important for defending against intracellular pathogens, such as viruses and certain bacteria, as well as for tissue graft rejection [16].

### 2.5 T Lymphocytes

T cells originate in the bone marrow and undergo maturation in the thymus. Lymphoid organs, such as the spleen and lymph nodes, receive T cells, which remain in a quiescent state until they are recognized by antigen-presenting cells [17]. T cells are involved in the initiation and maintenance of hypertension through cytokine production, activation, infiltration of the renal vasculature, and modulation of both the parasympathetic and sympathetic nervous systems.

T cells have a significant role in the development of hypertension. Mice deficient in T and B cells (RAG-1-/-) showed a diminished hypertensive response to both angiotensin II administration and DOCA-salt treatment, a model in which circulating angiotensin II levels are reduced [18]. Mice lacking CD8+ T cells were protected from endothelial impairment, renal salt and water retention, and blood vessel thinning induced by angiotensin II [19]. However, in RAG-1-/- mice, reintegrating CD8+ T cells, but not B cells, provokes sodium and fluid accumulation, reinstating the hypertensive response to these stimuli, indicating that CD8+ T cells may be essential for the hypertensive response [17]. Rats with a chromosomal alteration in the Rag1 gene, which leads to decreased levels of fully developed T and B cells, exhibit a notable reduction in salt-sensitive hypertension and renal disease [20]. Moreover, the significance of T cells in hypertension development is further supported by findings in mice with severe combined immune deficiency (SCID) and Dahl salt-sensitive rats with selective deletion of CD247, a gene crucial for T cell signalling. Both models are protected from hypertension despite exposure to hypertensive stimuli [17, 21].

### 2.6 T Cell Subsets

The most important components of the adaptive immune system are T lymphocyte subsets, which infiltrate injured tissues and eradicate invading microorganisms by secreting cytokines or cytotoxic factors. Four decades of research show extensive evidence that T lymphocyte subsets play a prominent role in blood pressure modulation. Their functions include antihypertensive effects in the kidneys, influencing sodium balance, contributing to endothelial dysfunction in blood vessels, regulating sympathetic nerve activity, and, more recently, actions in the skin that control interstitial sodium accumulation. Furthermore, salt consumption can have a direct impact on T cell differentiation [22].

CD3+ T lymphocytes can mainly be categorized into CD4+ or CD8+ single-positive cells. Both CD4+ and CD8+ T cells disperse from the thymus and attain immune competence. Activation requires two signals from antigen-presenting cells. The first signal is the activation of the TCR by an antigen presented on the MHC molecules of antigen-presenting cells. The second signal is co-stimulatory, usually provided through the interaction of specific molecules on APCs and the co-stimulatory molecule family on T cells. The CD4 and CD8 co-receptors on T cells interact with MHC class II and I, respectively. After primary stimulation, naive CD4+ T helper cells differentiate into various subsets, including Th1, Th2, Th17, T regulatory (Treg), Th9, or T follicular helper (Tfh) cells. CD8+ T cells differentiate into cytotoxic CD8+ T cells (Fig. **1**). Each differentiated T cell subset produces a distinct set of cytokines and carries out specific, specialized functions [22].

### 2.7 T Cell Binding Mechanism

In the thymus, a T cell develops a distinct antigen-recognizing protein on its surface known as the T cell receptor (TCR). Antigens are bound to cell-membrane proteins called major histocompatibility complex class II (MHC-II) molecules. They are recognized by TCRs (Fig. **2**). The T cell detects and interacts with the antigen–MHC class II complex and becomes an effector cell (activated) that produces a range of diverse growth factors collectively referred to as cytokines. The released cytokines are essential for activating B cells, Tc cells, macrophages, and various other cells involved in the immune response.

Upon presentation of an antigen within an MHC-II molecule to the TCR on a naive CD4+ T cell, the T cell differentiates into various cell phenotypes. These T cells produce different cytokines characterized by their distinct properties. The cytokines released by T cell subsets regulate nearby inflammatory cells in a paracrine manner, impacting both vascular reactivity and renal sodium handling [14, 23].

### 2.8 Activation of T lymphocytes in Hypertension

T cell induction necessitates the ligation of the T cell receptor (signal 1) and a co-stimulatory interaction between CD28 on T cells and B7 ligands on antigen-presenting cells (signal 2). Vinh *et al.* [24] demonstrate that inhibiting these co-stimulatory interactions, either through the use of an inhibitor or genetic deficiency of the B7 ligands, reduces hypertension and restricts perivascular T cell infiltration. Similarly, altering TCR ligation by mutating a TCR signaling molecule diminishes the hypertensive response to a salt-loaded diet in the Dahl salt-sensitive rat model [21]. Thus, both signal 1 and signal 2 are essential for the initiation of hypertension.

A slight increase in blood pressure serves as a trigger for T cell activation. This causes inflammatory cells to migrate into the kidney, where they remain sensitive to salt even after the hypertensive stimulus is withdrawn [25]. Marvar *et al.* [26] showed that either blocking sympathetic outflow from the CNS or administering hydralazine prevents T cell activation and perivascular infiltration, thereby stopping blood pressure elevation in angiotensin II–infused mice. Highly reactive gamma-ketoaldehydes (isoketals) induce oxidative stress in antigen-presenting cells, boosting their capacity to activate T cells and promoting susceptibility to hypertension. A high-salt diet can contribute to the development of hypertension not only by expanding intravascular volume but also by promoting Th17 differentiation through the MAPK signaling pathway or the inducible salt-sensing kinase SGK1 [27, 28].

### 2.9 CD4+ T Helper (Th) Lymphocyte

CD4^+^ T helper cells, subclassified into Th1, Th2, Th17, Treg, Th9, and Tfh, secrete specific cytokines and perform distinct functions [22]. Th1 cells produce their signature cytokines, IFN-γ, TNF-α, and IL-2 [29], through the action of the transcription factor T-bet, which plays a prominent role in the progression of hypertension [18, 30].

### 2.10 Tumor Necrosis Factor - α

Tumor Necrosis Factor Alpha (TNF-α) is a multi-faceted, proinflammatory peptide cytokine that also has anti-tumorigenic properties and is released by lymphocytes, macrophages, endothelial cells, and epithelial cells [31-33]. The source of TNF-α in the kidney includes epithelial cells from the proximal tubules, thick ascending limbs, and collecting ducts [34, 35]. Activation of the calcium-sensing receptor (CaR) stimulates Gi and Gq (G protein-coupled receptors), induces stimulation of phosphatidylinositol-specific phospholipase C (PLC), and provokes the generation of inositol triphosphate (IP3) and diacylglycerol (DAG). IP3 enhances intracellular Ca2+ levels and forms a complex with calmodulin, while DAG activates protein kinase C (PKC), triggering the activation of calcineurin. Dephosphorylation of nuclear factor of activated T cells (NFAT) by calcineurin causes translocation of this transcription factor to the nucleus and transcription of TNF-α [36-38] (Fig. **3**). Upon administration of ANG II to TNF-α knockout mice, the blood pressure increase failed, but it was restored with substitution therapy using recombinant TNF-α (0.3 g/kg/day) [34, 39].

Extraneous administration of angiotensin II or its production *in vivo* increases T cell activity, which in turn elevates TNF-α, and blockade of TNF-α attenuates blood pressure and reduces vascular oxygen production. This implies a dual role of T cells by elevating CD44, CCR5, and RANTES in vessels. The latter favor the vascular accumulation of T cells, especially CD4+ and, to a lesser extent, CD8+ T cells in the adventitia and periadventitial fat of hypertensive animals [18, 34]. NFκB plays a pivotal role in the progression of acute and chronic inflammatory disease by transcribing genes that coordinate responses to pathogens and proinflammatory cytokines. The binding of NFκB to its inhibitory protein, IκB, in the cytoplasm results in the formation of a p50-p65 heterodimer. Proinflammatory cytokines, such as TNF-α, stimulate IκB kinases (IKKs), which phosphorylate IκBα, thereby activating NF-κB [40]. TNF-α reduces renin-promoting activity *via* NFκB. Todorov *et al*. [41] suggested that TNF-α actions depend on NF-κB binding to the cAMP response element (CRE), which is present on the renin promoter for regulating renin expression [34, 41]. ANG II–induced hypertension is characterized by monocyte expansion and kidney infiltration, accompanied by increased production of the proinflammatory cytokine TNF-α by T lymphocytes. Exogenous TNF administration enhances natriuresis by binding to TNF receptor 1 (TNFR1). TNF-α induces increased macrophage infiltration, leading to lymphocyte activation, enhanced renal NaCl reabsorption, kidney dysfunction, and proteinuria. However, during rapid stimulation of the renin–angiotensin system (RAAS), endogenous TNF contributes to increased blood pressure and kidney injury [34, 42, 43].

### 2.11 Interferon-γ

CD4+ T cells, CD8+ T cells, and gamma delta T cells (γδT cells) produce interferon-gamma (IFN-γ) [44, 45]. Macrophages also produce IFN-γ, which regulates Th1 differentiation and activates myeloid cells and B lymphocytes. IFN-γ controls sodium elimination in urine by activating the sodium/hydrogen exchanger 3 (NHE3) transporter in the proximal convoluted tubule (Fig. **4**) and by increasing sodium-potassium-chloride co-transporter 2 (NKCC2) and sodium-chloride co-transporter (NCC) activity in the distal convoluted tubule [46]. Inhibition of IFN-γ receptor 1 (IFNR1) prevents the development of tubular and interstitial inflammation during Ang II–dependent hypertension [47]. Benson *et al*. [48] concluded that IFN-γ knockout results in decreased hypertension and reduced accumulation of renal T cells caused by DOCA salt administration in mice. Similarly, knockdown of PDL1 expression using siRNA encapsulated in kidney tubule-specific nanoparticles reduces hypertension and diminishes renal cell accumulation in salt-sensitive mice. Therefore, the angiotensin II and DOCA salt models show a blunted blood pressure response in IFN-γ knockout mice [46, 48]. Thangaraj *et al*. [49] demonstrated that plasma levels of IFN-γ and IL-6 are diminished by spironolactone. The antihypertensive effect of spironolactone in resistant hypertension is linked to the suppression of IFN-γ and IL-6, but not IL-17A [50]. A decrease in IFN-γ levels in models of hypertension or cardiac failure leads to diminished macrophage migration and IL-6 signaling, reduced T cell infiltration into tissues, enhanced cardiac performance, and improved capillary distribution, along with better cell junction integrity in the cerebrovasculature [45]. Ishimitsu *et al*. [51] suggested that IFN-γ lowers hypertension, reduces proteinuria, and mitigates glomerular impairment in salt-sensitive Dahl rats; it also hinders the proliferation of vascular smooth muscle cells and endothelial cells. The renal arterioles of hypertensive animals show deposition of immunoglobulins or infiltration of mononuclear cells, especially in malignant salt-induced hypertension.

### 2.12 Interleukin-10

Interleukin-10 (IL-10) is synthesized by Th2 lymphocytes [22], Tregs, mast cells, and monocytes, and is an important anti-inflammatory cytokine [3, 52, 53]. In its inactive form, NF-κB binds to the inhibitory protein IκB, which exists in the cytoplasm as a p50-p65 heterodimer (Fig. **5**). Cytokines like TNF-α activate NF-κB by phosphorylating IκB *via* IκB kinases (IKK). This causes the release of NF-κB and its transport to the nucleus, leading to gene transcription. IL-10 suppresses the release of proinflammatory cytokines and NF-κB, which ultimately controls inflammation [40]. Exogenous IL-10 administration in rats reduces proteinuria, endothelial injury, and elevated blood pressure in pregnancy-induced hypertension [54]. Angiotensin II augments superoxide radicals in the vasculature of IL-10-deficient mice, illustrating the protective effects of endogenous IL-10 against oxidative stress even at low concentrations. Ang II elevates superoxide in blood vessels of control mice and induces NADPH oxidase-dependent endothelial impairment [55]. Proinflammatory cytokines such as IL-6, TNF-α, and NF-κB are inhibited by IL-10 [55-57]. Ang II elevates vascular IL-6 expression and TNF-α in normal mice while decreasing IL-10 levels, which further increases IL-6 mRNA expression. Thus, IL-10 regulates IL-6 levels, endothelial dysfunction, and reactive oxygen species in response to Ang II [55]. IL-10 attenuates NF-κB DNA binding triggered by

TNF-α and LPS in a dose-dependent manner, specifically impacting the p65/p50 heterodimer complex. IL-10 blocks the cellular signaling pathway required for NF-κB DNA binding activity [40]. Gunnett *et al*. [58] illustrated that IL-10 deficiency contributes to increased superoxide production and endothelial dysfunction, whereas IL-10-/- mice show attenuation of superoxide elevation and endothelial dysfunction during inflammation.

### 2.13 Th17 Lymphocyte

Much like Th1 cells, Th17 cells produce several proinflammatory interleukins, including IL-17, IL-21, and IL-22, which play a crucial role in combating external pathogens and contributing to autoimmune conditions. Th17 cells and IL-17 have various functions, including regulating sodium–water balance in the kidneys and coordinating endothelial cell dysfunction, ultimately leading to exacerbated increases in blood pressure [59, 60].

### 2.14 Interleukin-17

Interleukin-17 (IL-17) and its receptors play a pivotal role in the body’s defense against pathogenic microorganisms. There are six known IL-17 family members, IL-17A–F, which possess potent proinflammatory activity and are produced by various immune cells [61, 62]. In response to immune activation, IL-17 is synthesized by dendritic cells, macrophages, NK cells, NKT cells, and γδT cells [62, 63]. Increased levels of IL-17 are associated with hypertension and autoimmune disorders linked to hypertension, such as pre-eclampsia, systemic lupus erythematosus (SLE), and chronic allograft rejection. During inflammation, IL-17 stimulates the production of cytokines and chemokines, driving the proinflammatory response and facilitating migration of immune cells to the site of infection [63]. IL-6, TNF-α, and IL-1β production is stimulated by IL-17, which is also associated with endothelial dysfunction and hypertension [63-65]. Induction of NADPH oxidase activity and enhanced superoxide production are mediated by IL-17 and Rho kinase. IL-17 promotes RhoA/Rho-kinase–mediated phosphorylation of eNOS at Thr495, resulting in reduced NO-dependent vasodilation and contributing to the onset of hypertension in mice (Fig. **6**). Administration of exogenous IL-17 worsens hypertension and endothelial dysfunction through activation of the Rho-kinase pathway [63]. Deficiency or blockade of IL-17A, but not IL-17F, reduces blood pressure elevation and kidney inflammation in Ang II-dependent hypertension in mice [59]. Additionally, removal of γδ T cells, which serve as a supplementary source of IL-17, markedly reduces the Ang II-dependent increase in blood pressure and vascular damage [12].

### 2.15 Treg Lymphocyte

Around 10% of CD4+ T cells are Treg cells. These cells suppress both innate and adaptive immune responses by releasing anti-inflammatory cytokines such as IL-10, IL-35, and TGFβ1. Treg cells are believed to influence hypertension primarily through IL-10, which is acknowledged as a crucial cytokine in restoring endothelial function and regulating blood pressure [66]. Chen *et al*. [67] demonstrated the roles of the complement system and T regulatory cells in the pathogenesis of hypertension. In hypertensive patients, C3a and C5a levels were increased, and Tregs from these patients had elevated C5aR expression. Activation of C3aR and C5aR reduces Treg function and promotes Ang II–induced hypertension. Therefore, blocking the complement system to preserve Treg function may be protective and serve as a therapeutic strategy for hypertension.

### 2.16 Transforming Growth Factor-β

Transforming Growth Factor-β (TGF-β): TGF-β1 or TGF-β2 induces vascular dysfunction and increases sodium retention, leading to elevated blood pressure [68]. TGF-β is a versatile cytokine with profibrogenic effects, contributing significantly to the development of cardio-renal and vascular end-organ damage associated with hypertension. Long-term administration of an anti-TGF-β antibody (murine monoclonal antibody 1D11) in Dahl salt-sensitive rats fed a high-salt diet was found to reduce mean arterial pressure, proteinuria, and renal medullary interstitial fibrosis. The murine monoclonal antibody 1D11 targets all three forms of TGF-β (TGF-β1, TGF-β2, and TGF-β3), significantly attenuating blood pressure elevation and glomerulosclerosis. These findings suggest that prolonged administration of an anti-TGF-β antibody in Dahl S rats significantly lowers blood pressure, proteinuria, and albuminuria [69, 70]. Conversely, TGF-β produced by T regulatory lymphocytes (Tregs) works together with IL-10 to mitigate hypertensive responses by inhibiting the activation of T effector lymphocytes [71].

### 2.17 Cytotoxic CD8+ T lymphocyte

CD8+ T cells become activated and differentiate into cytotoxic T cells. They bind to MHC-I and produce cytokines such as IFN-γ, TNF-α, granzyme B, and perforin. These cytokines contribute to cell apoptosis and inflammation. Trott *et al*. [19] demonstrated that administration of angiotensin II/DOCA salt to CD8-/- mice resulted in blunted hypertension and studied the effect of sodium volume retention *via* intraperitoneal injection of physiological saline equivalent to 10% of their body weight alongside angiotensin II infusion. IFN-γ produced by CD8+ T cells accumulates in the hypertension-challenged kidney. CD8+ T cells play a central role in mediating angiotensin II–induced endothelial dysfunction, vascular contraction, and disruption of water–sodium balance, all of which are essential factors in the progression of hypertension. CD8+ T cell–mediated vascular contraction leads to Na+ retention and fluid expansion. Thus, cytotoxic CD8+ T cells aggravate vascular tone, resulting in elevated blood pressure.

Sun *et al*. [72] described a novel cellular pathway associated with CD8+ T cell activation in hypertension. Mineralocorticoid receptor (MR) expression on CD8+ T cells triggers the production of pro-hypertensive cytokines mediated by nuclear factor of activated T cells 1 (NFAT1). Administration of angiotensin II in T cell MR knockout hypertensive mouse models demonstrates that MR deficiency in T cells decreases both systolic and diastolic blood pressure and mitigates kidney and vascular damage. This effect is evidenced by reduced albuminuria, IFN-γ levels, and Ang II–induced renal fibrosis, indicating improved renal function. Therefore, CD8+ T cells play a central role in the development of hypertension.

### SUMMARY AND CONCLUSION

As a consequence, immune cells become activated, leading to vasoconstriction and sodium–water retention in the kidney. Several recent studies have provided evidence that inflammatory cytokines play a significant role in mediating hypertension. A few scientific experiments have demonstrated the role of immune cells and their cytokines in the development of hypertension. This review aims to illustrate the role of T cells in alterations of vascular reactivity, endothelial dysfunction, sodium–water excretion, and immune cell infiltration observed in hypertension.

In conclusion, substantial evidence indicates that T cells and their cytokines play a pathophysiological role in the progression of hypertension and the triggering of end-organ dysfunction. There is a need to focus on the pharmacological mechanisms to improve the disease condition. Anti-inflammatory and immunosuppressive drugs are not currently used to treat hypertension; however, balanced modulation of the immune system may be necessary to prevent organ damage without suppressing immune function. Additional investigations are needed to evaluate these drugs as adjuvant therapy alongside antihypertensive medications. Conventional antihypertensive drugs are used to control hypertension and protect target organs, while novel and insightful immunotherapies are still in development. Further research is required to assess antihypertensive drugs that may specifically target T cell functions and cytokine activity. Medications developed for other disease conditions may also target proteins or pathways involved in hypertension and/or reduce circulating cytokines, such as TNF-α, IFN-γ, and IL-17. These drugs warrant further investigation to determine their potential benefits in hypertension.

## Figures and Tables

**Fig. (1):**
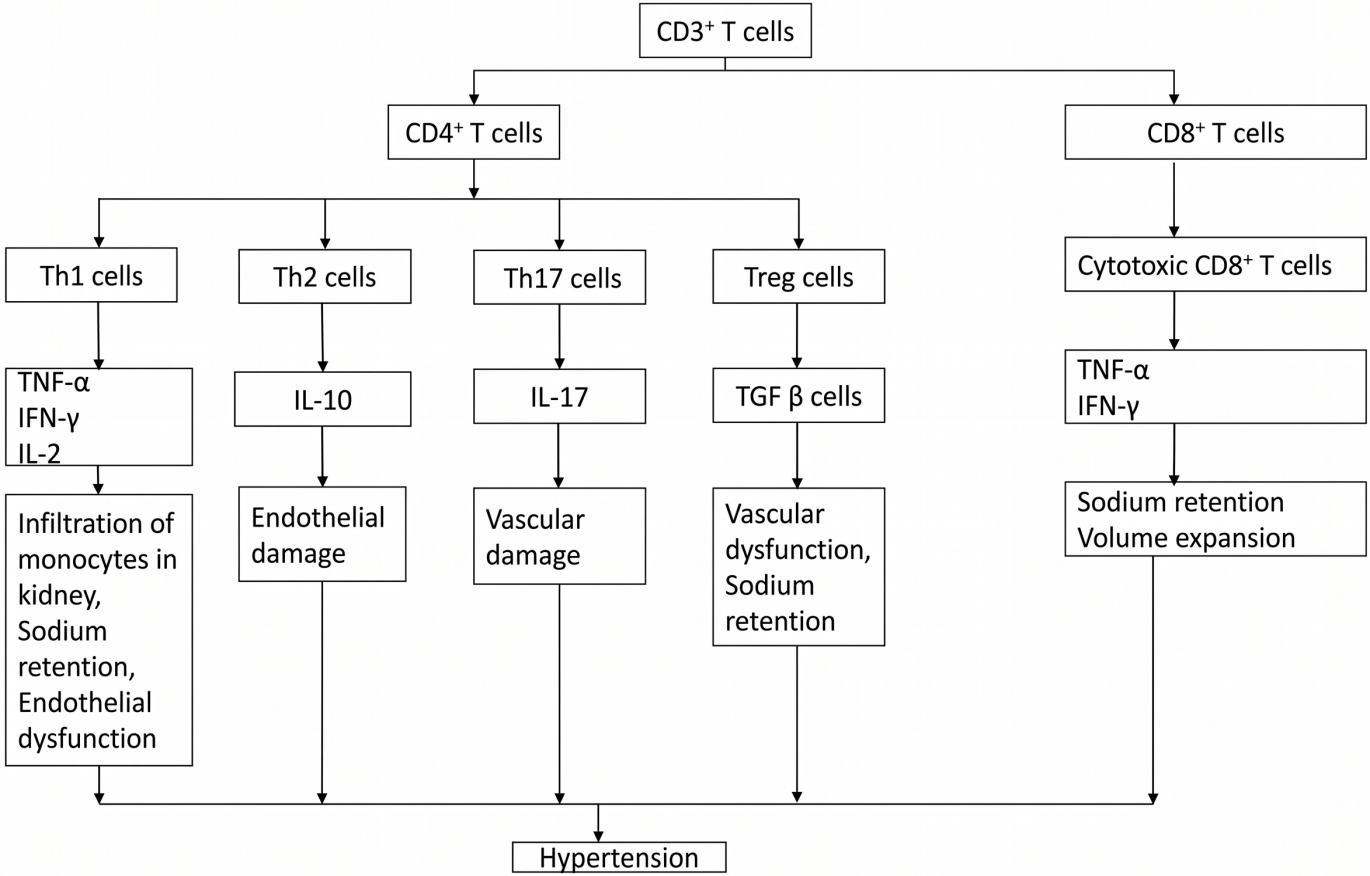
Classification of T cells and their cytokines, along with their action in hypertension.

**Fig. (2):**
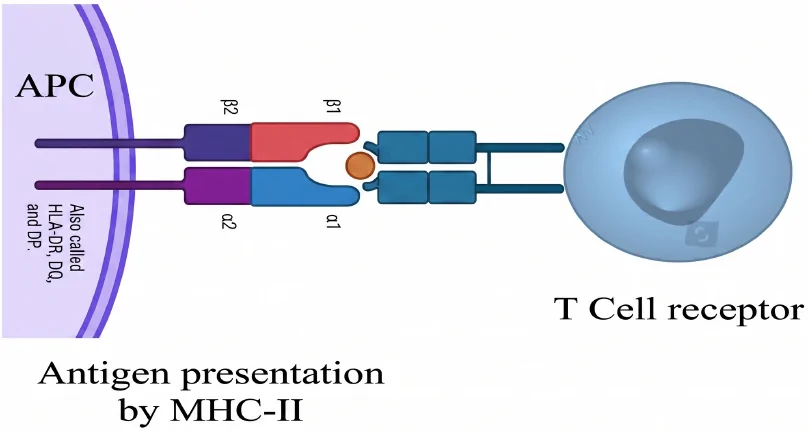
Antigen-presenting cells expressing the MHC-II molecule present the antigen, which binds to the T cell receptor.

**Fig. (3):**
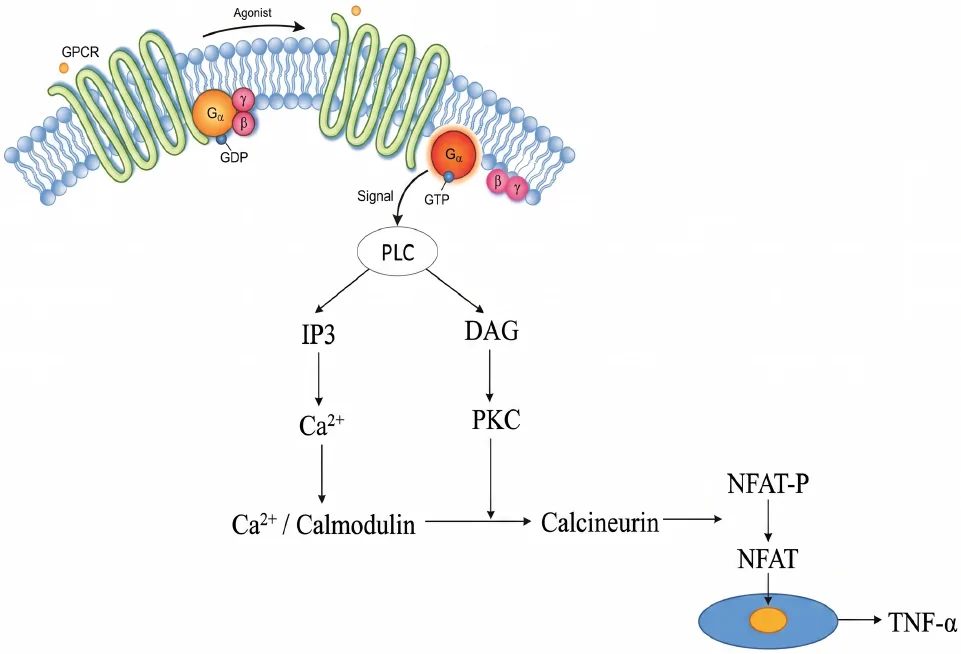
Synthesis of Tumor Necrosis Factor (TNF-α) *via* G Protein Coupled Receptor (GPCR) mechanism.

**Fig. (4):**
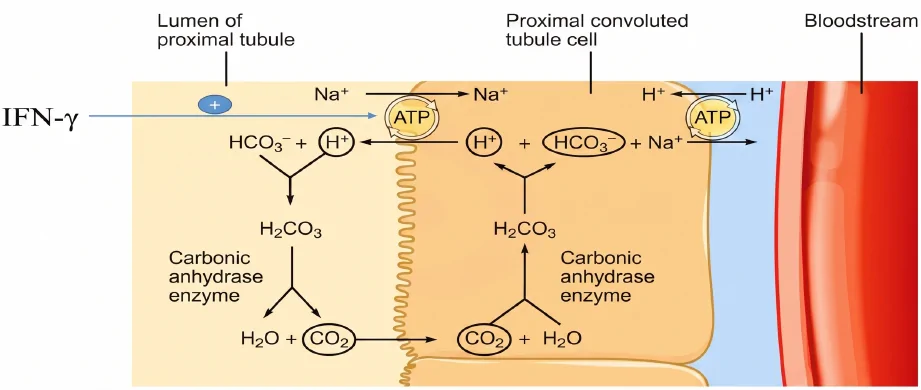
Action of Interferon-γ (IFN-γ) in Na^+^/H^+^ exchanger 3 (NHE3) present in proximal convoluted tubule.

**Fig. (5):**
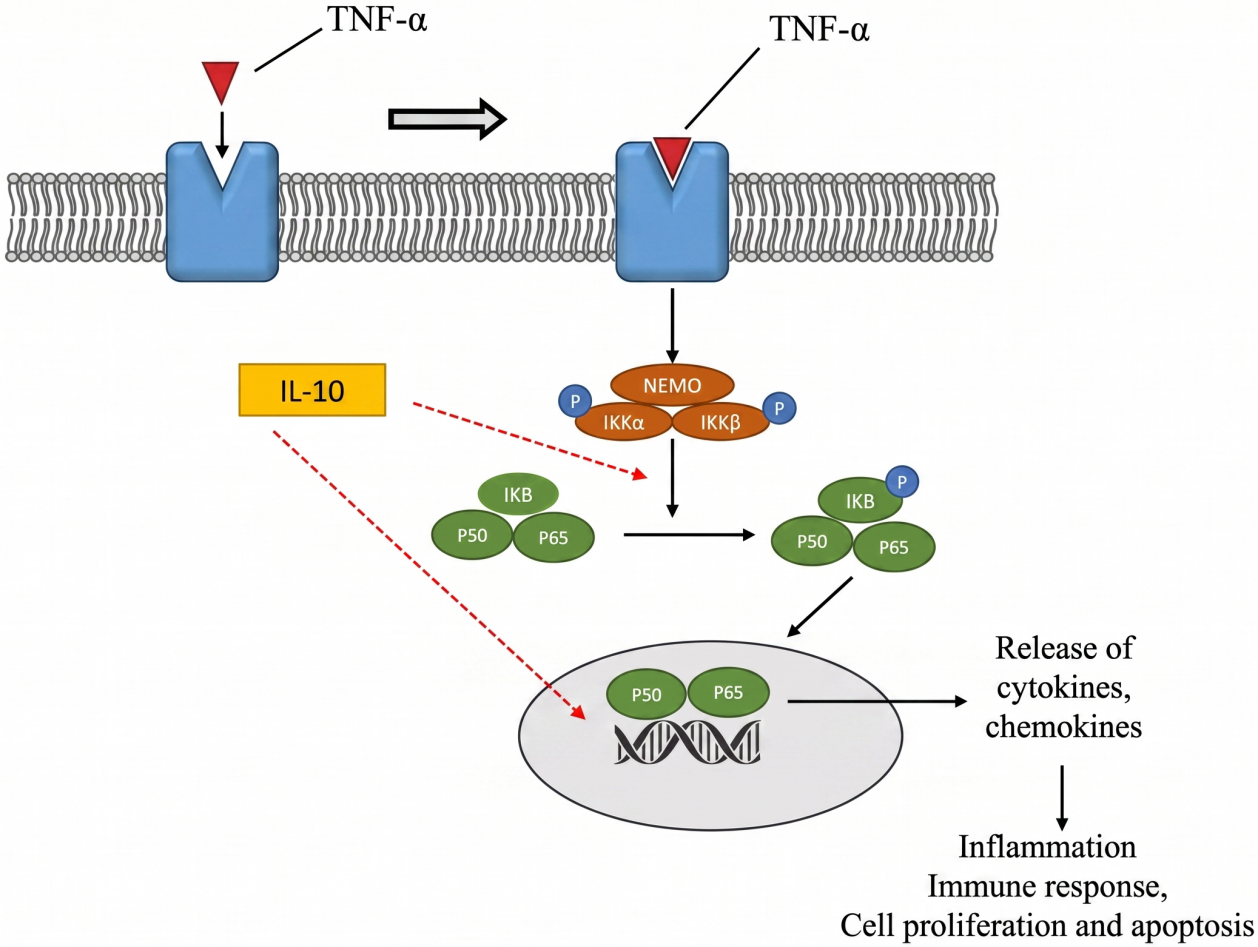
Action of Interleukin – 10 (IL-10) in inhibiting Nuclear Factor kappa B (NF-κB), which eventually causes inflammation.

**Fig. (6):**
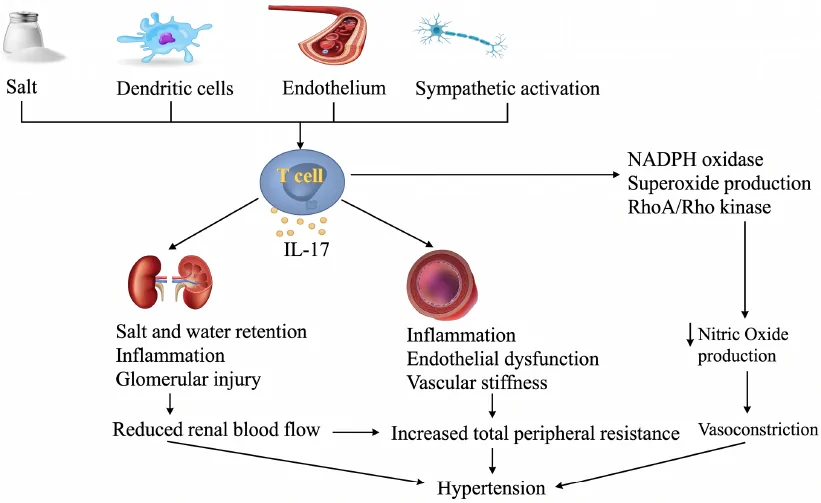
Action of Interleukin-17 (IL-17) in the kidney and blood vessel that leads to hypertension.

## LIST OF ABBREVIATIONS

Htn: Hypertension
Bp: Blood Pressure
Ang-Ii: Angiotensin Ii
Et-1: ndothelin
Igg: Immunoglobulin G
Ros: Reactive Oxygen Species
Prrs: Pattern Recognition Receptors
TLRs: Toll-Like Receptors
Pamps: Pathogen-Associated Molecular Patterns
Damps: Damage-Associated Molecular Patterns
Doca: Deoxycorticosterone Acetate
SCID: Severe Combined Immune Deficiency
MHC: Major Histocompatibility Complex
Tcr: T Cell Receptor
Mapk: Mitogen-Activated Protein Kinase
Ifn Γ: Interferon Γ
Tnf Α: Tumor Necrosis Factor Α
IL-2: Interleukin 2
Car: Calcium Sensing Receptor
Plc: Phospholipase C
IP3: Inositol Triphosphate
Dag: Diacylglycerol
Pkc: Protein Kinase C
NFAT: Nuclear Factor Of Activated T Cells
CCR5: C-C Chemokine Receptor 5
Rantes: Regulated Upon Activation
Cd44: Cluster Of Differentiation 44
Nfkb: Nuclear Factor K Beta
Cre: Camp Responsive Element
Tnfr1: Tumor Necrosis Factor Receptor 1
Raas: Renin Angiotensin Aldosterone System
Nhe3: Sodium Hydrogen Exchanger 3
Nkcc2: Sodium Potassium Chloride Cotransporter 2
Ncc: Sodium Chloride Cotransporter
NADPH: Nicotinamide Adenine Dinucleotide Phosphate
LPS: Lipopolysaccharide
Nk Cells: Natural Killer Cells
C3a And C5a: Complement Component 3a and 5a
